# Metabolomic biomarkers in autism: identification of complex dysregulations of cellular bioenergetics

**DOI:** 10.3389/fpsyt.2023.1249578

**Published:** 2023-10-02

**Authors:** Alan M. Smith, Elizabeth L. R. Donley, Denise M. Ney, David G. Amaral, Robert E. Burrier, Marvin R. Natowicz

**Affiliations:** ^1^Stemina Biomarker Discovery, Inc, Madison, WI, United States; ^2^Department of Nutritional Sciences, University of Wisconsin-Madison, Madison, WI, United States; ^3^Department of Psychiatry and Behavioral Sciences, The MIND Institute, University of California, Davis, Davis, CA, United States; ^4^Pathology and Laboratory Medicine, Genomic Medicine, Neurological and Pediatrics Institutes, Cleveland Clinic, Cleveland, OH, United States

**Keywords:** autism, pathophysiology, bioenergetics, subtypes, biomarker, metabolomic, stratification, mitochondria

## Abstract

Autism Spectrum Disorder (ASD or autism) is a phenotypically and etiologically heterogeneous condition. Identifying biomarkers of clinically significant metabolic subtypes of autism could improve understanding of its underlying pathophysiology and potentially lead to more targeted interventions. We hypothesized that the application of metabolite-based biomarker techniques using decision thresholds derived from quantitative measurements could identify autism-associated subpopulations. Metabolomic profiling was carried out in a case–control study of 499 autistic and 209 typically developing (TYP) children, ages 18–48 months, enrolled in the Children’s Autism Metabolome Project (CAMP; ClinicalTrials.gov Identifier: NCT02548442). Fifty-four metabolites, associated with amino acid, organic acid, acylcarnitine and purine metabolism as well as microbiome-associated metabolites, were quantified using liquid chromatography-tandem mass spectrometry. Using quantitative thresholds, the concentrations of 4 metabolites and 149 ratios of metabolites were identified as biomarkers, each identifying subpopulations of 4.5–11% of the CAMP autistic population. A subset of 42 biomarkers could identify CAMP autistic individuals with 72% sensitivity and 90% specificity. Many participants were identified by several metabolic biomarkers. Using hierarchical clustering, 30 clusters of biomarkers were created based on participants’ biomarker profiles. Metabolic changes associated with the clusters suggest that altered regulation of cellular metabolism, especially of mitochondrial bioenergetics, were common metabolic phenotypes in this cohort of autistic participants. Autism severity and cognitive and developmental impairment were associated with increased lactate, many lactate containing ratios, and the number of biomarker clusters a participant displayed. These studies provide evidence that metabolic phenotyping is feasible and that defined autistic subgroups can lead to enhanced understanding of the underlying pathophysiology and potentially suggest pathways for targeted metabolic treatments.

## 1. Introduction

Autism Spectrum Disorder, a condition with marked etiological and clinical heterogeneity, has a prevalence of over 2% in the United States and is associated with considerable personal, family, and societal challenges (1–4). As autism remains a behaviorally defined condition, there have been extensive efforts to understand its underlying cellular and molecular bases and to discover clinically useful biomarkers (1, 2). There have been multiple efforts to stratify autism using molecular and behavioral-based endpoints (5–7). Identifying biochemical subtypes may provide a path to stratification that can lead to earlier diagnosis and more effective treatments (5, 6, 8–10).

A range of biomarker modalities for the screening of autism have been investigated including genomic, transcriptomic, proteomic, neuroimaging, EEG, eye tracking and metabolic markers (11–14). There has been substantial interest in exploring metabolic underpinnings of autism from the dual perspectives of yielding pathophysiologic insights and in discovering biomarkers for more precise treatment. Previous studies have reported many potential metabolic alterations to be associated with autism (15–21). However, few of the biomarkers have been replicated (14). It is likely that the lack of generalizability for the majority of autism-related biomarkers is due to small sample sizes, autism heterogeneity, and other study design issues (14, 22–24). We conducted the multicenter Children’s Autism Metabolome Project (CAMP, ClinicalTrials.gov Identifier: NCT02548442) to recruit a large number of children, ages 18–48 months, and used metabolomics-specific protocols to identify biomarkers and metabolic phenotypes associated with autism.

Metabolic phenotypes are biochemical signatures that reflect an individual’s unique metabolism and result from the interplay of one’s genetic background, environment, microbiome, co-occurring conditions, and diet (25). Due to the clinical and etiological heterogeneity of autism, distinct metabolic subpopulations of autism will likely have low prevalence. Therefore, metabolic tests based on biomarkers that identify autism-associated metabolic subpopulations will require sensitivities that detect low prevalence metabolic phenotypes, have high specificities to distinguish the phenotype and, ideally, provide new or support existing biological insights.

The current study further explores the hypothesis that the application of metabolite-based biomarker techniques using decision thresholds derived from quantitative measurements can identify metabolic subpopulations of autistic individuals (6, 9, 10). Our earlier metabolic phenotyping work provides support for this vision (16, 26). We now extend that work by evaluating additional metabolites and ratios of metabolites, especially ones related to the microbiome and cellular bioenergetics. The evaluation of these metabolites and ratios uncovered biologically plausible biomarkers that expand upon the biochemical processes associated with the pathophysiology of autism.

## 2. Materials and methods

### 2.1. Children’s autism metabolome project participants

The Children’s Autism Metabolome Project (CAMP, ClinicalTrials.gov Identifier: NCT02548442) study enrolled 1,102 children, ages 18–48 months, across 8 clinical sites from August, 2015 through January, 2018. We selected this age range because a consensus has emerged that a professional diagnosis of autism can be carried out accurately as early at 18 months of age. The centers included: The Children’s Hospital of Philadelphia; Cincinnati Children’s Hospital; The Lurie Center at Massachusetts General Hospital; The Melmed Center; The MIND Institute, University of California – Davis; Nationwide Children’s Hospital; The University of Arkansas for Medical Sciences; and Vanderbilt University Medical Center. Written informed consent from a parent or legal guardian was obtained and monetary compensation was provided to each participant. The study protocol was approved and monitored by Institutional Review Boards at each of the clinical centers.

#### 2.1.1. Participant clinical and parental assessments

Each participant underwent physical and neurological examinations and behavioral testing performed by clinicians. Parental interviews and medical records were used to obtain each participant’s age, race, medications, and dietary information, as well as prenatal, perinatal, medical, and developmental histories.

#### 2.1.2. Behavioral testing and diagnosis

The Autism Diagnostic Observation Schedule-Second Version (ADOS-2) assessment (27) was performed by research reliable clinicians on CAMP participants enrolled with a suspected diagnosis of autism. CAMP participants were classified as autistic if the ADOS-2 Module-1 or Module-2 Comparison Score (CS) was greater than 3 or an ADOS-2 Toddler Module Range of Concern was designated Mid-to-moderate or Moderate-to-severe. ADOS-2 comparison severity scores (CSS) were calculated for the Social Affect (SA) and Restrictive, Repetitive Behavior (RBB) scores for participants administered Module-1 or Module-2 (28). CSS scores were not calculated for participants administered the Toddler Module due to missing language ability information required to calculate the CSS (29). The Mullen Scales of Early Learning (MSEL) (30) was administered to all children enrolled in CAMP and used to derive a developmental quotient (DQ) based on the composite standard score. CAMP participants were considered typically developing (TYP) if the MSEL DQ was greater than 70 and the participant did not receive a diagnosis of developmental delay or autism. Only subjects with a confirmed diagnosis of ASD or TYP were included in this study.

#### 2.1.3. Exclusion criteria

Enrollment was limited to one child per household to minimize genetic or family environmental effects. Children participating in other clinical studies could not have used any investigational agent within 30 days of participation. Children were excluded from the study if they were previously diagnosed with a genetic condition such as Fragile X syndrome, Rett syndrome, Down syndrome, tuberous sclerosis, or inborn errors of metabolism. Participants with fetal alcohol syndrome, serious neurological disorders, metabolic, psychiatric, cardiovascular, or endocrine system disorders were also excluded. Participants exhibiting acute signs of illness within 2 weeks of enrollment such as vomiting, diarrhea, fever, cough, or ear infection were rescheduled.

### 2.2. Phlebotomy and preanalytical specimen handling procedures

Blood was collected from participants who had not eaten for at least 12 h by venipuncture into 6 mL sodium heparin tubes placed on wet ice (16, 26). Plasma was obtained after centrifugation (1,200 g for 10 min at room temperature) and stored at −80°C within 60 min of the blood draw. Hemolysis of samples was measured using spectrophotometry of the plasma (31). Plasma from hemolyzed samples with hemoglobin >600 mg/dL were excluded from metabolomics analyses and concentration values for xanthine, uric acid, or hypoxanthine were omitted when hemoglobin exceeded 300 mg/dL (26).

### 2.3. Quantitative liquid chromatography—tandem mass spectrometry analysis

Three quantitative LC–MS/MS methods measuring 54 small molecule metabolites were performed in a CLIA-certified laboratory. The methods were analytically validated in compliance with FDA and CLSI guidance for bioanalytical method validation (32, 33). Quantification of analytes was performed using an Agilent Technologies G6490 triple quadrupole mass spectrometer. Detailed information about the sample preparation, detection, and quantification of metabolites can be found in the Supplemental Data Sheet. Analyte measurements below the lower limit of quantification (LLOQ) or above the upper limit of quantification (ULOQ) values were replaced with 90% of the LLOQ or 110% of the ULOQ value.

### 2.4. Metabolomics participant sample set

CAMP enrolled 1,102 participants and 916 met the inclusion and exclusion criteria described above (Supplementary Figure 1). Of these, 608 received a diagnosis of autism and 214 were considered TYP. The participant sample set was established after removing 32 autistic and 4 TYP samples that were hemolyzed, 77 autistic and 1 TYP participants’ samples that failed LC–MS/MS acquisitions, and 94 participants with developmental delay (DD) without autism. The final sample set contained 708 participant samples from 499 autistic and 209 TYP children.

### 2.5. Metabolomic data analysis

We measured the concentrations of 54 metabolites and also evaluated the ratios of these metabolites (Supplementary Table 1). Metabolite ratio analysis can detect changes or reveal biological processes that may not be discerned by individual metabolites (34). For example, the concentrations of metabolites in a metabolic reaction sequence that has a minimal, but physiologically relevant, alteration of function of an enzyme or transporter may not show apparent alterations of the metabolites of that pathway. However, if the concentration of a metabolite that is distal to the metabolic reaction is expressed as a ratio to a metabolite that is proximal to the metabolic reaction, that ratio may reveal a change in that pathway that could otherwise go undetected. In addition, ratios of the concentration of metabolites can provide a normalization effect that reduces variation due to unrelated biological or analytical sources leading to higher specificity in diagnostic analyses (34). Clinical applications of metabolite ratio analysis include use in newborn screening for some inherited disorders of amino acid and organic acid metabolism and of mitochondrial fatty acid beta-oxidation (35, 36). Because of the benefits of ratio analysis, metabolite ratios are also utilized in association analyses with genetic variants and phenotypes to identify the metabolic underpinnings of more complex, multifactorial biological processes (37–39).

The metabolite ratios were generated from all unique combinations of the metabolites except 3-carboxy-4-methyl-5-propyl-2-furanpropanoic acid (CMPF), 4-ethylphenylsulfate (4-EPS) and dodecanedioic acid where 90% of the measurements were below the LLOQ. To create uniform visualization of metabolite ratios, the numerator and denominator were selected to yield a ratio with values that are greater than the biomarker threshold (see Biomarker Analysis). The concentrations of each metabolite or ratio of metabolites values were log base 2 transformed to reduce skewness and standardized (μ = 0, σ = 1) by subtracting the mean and dividing by the standard deviation prior to analyses. Participants’ metabolite measurements with missing data were omitted from analysis reducing the number of samples analyzed for a test statistic or imputed with the median value when missing values are not allowed by a test statistic. Analyses were conducted using R version 4.1.0 (40).

### 2.6. Biomarker analysis

**Figure 1:**
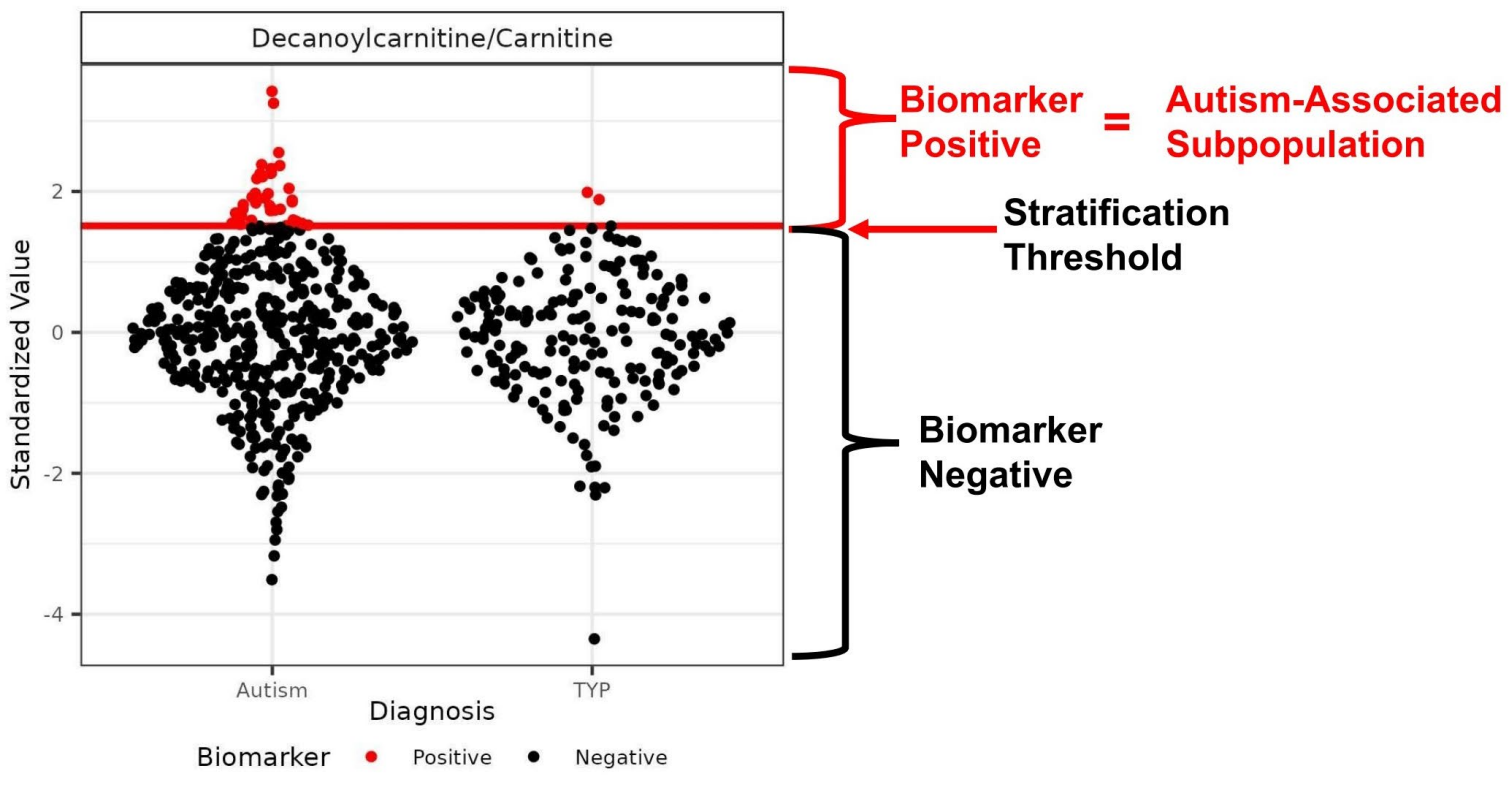
Example of an autism-associated biomarker of a metabolic subpopulation. Scatter plot of the ratio of the metabolites decanoylcarnitine/carnitine with the threshold used to create a subpopulation of autistic individuals that is largely distinct from the TYP population. The threshold is represented as a red horizontal line used to separate the CAMP population into biomarker-positive (red) and biomarker-negative (black) participants. The threshold is set to maximize the percentage of autistic individuals in the subpopulation, maintaining a minimum of 4.5% of the CAMP autistic population above the threshold. In this example, the autism-associated subpopulation contains 7.3% of the CAMP autistic population and 1% of the TYP population; the proportion of autistic individuals (PMP-ASD) is 95%.

Receiver operator curve (ROC) analysis was used to select a biomarker value threshold (Figure 1) that maximized the percentage of autistic participants at a sensitivity above 4.5% when exceeded (26). The performance metrics were estimated using 4-fold cross-validation, repeated 50 times, stratified by participant sex, age, and diagnosis. This method of cross-validation trains and tests a model four times using independent sample sets, based on a training set of 75% and a test set of 25% of the samples, with model performance assessed as the average test set performance across repeats. Metabolites and ratios of metabolites were considered an autism-associated biomarker if the average performance had a sensitivity of at least 4.5% (indicating a subpopulation of at least 4.5% of the autism study participants that were biomarker-positive) and the proportion of the biomarker-positive (PMP) population of at least 90% autistic individuals (equivalent to the positive predictive value (PPV) of 90% within the CAMP study population prevalence). In addition to the sensitivity and PMP criteria, the permutation-based test statistic was significant at a false discovery rate adjusted *p*-value <0.1 (16, 26).

The final model thresholds were set using the entire study set of CAMP autism and TYP participants. These thresholds were used to generate participant biomarker outcome profiles by scoring a participant positive or biomarker negative for each biomarker. CAMP participant biomarker profiles were used to cluster the biomarkers, determine the prevalence of biomarker clusters in CAMP, and comparisons based on biomarker positive and negative populations. An overview of the methods used for biomarker selection and creating participant biomarker profiles is presented in Supplementary Figure 2. Optimization of biomarkers for identification of a likelihood of autism was performed using the process described previously (26).

### 2.7. Clustering analysis

Clustering was performed to reduce the complexity of the biomarkers by aggregating related biomarkers or biomarker clusters into groups. Hierarchical complete-linkage clustering of the participant biomarker outcome profiles was performed using the Jaccard distance based on scoring a biomarker outcome as 0 = negative and 1 = positive. The optimal number of biomarker clusters was estimated using the maximum value of the average silhouette width cluster validation index over a range of 5 to 50 clusters. The biomarker clusters were further evaluated by clustering the fold changes of metabolites by hierarchical complete-linkage clustering using a distance matrix based on the Pearson correlation coefficients (|1-r|) of the metabolite fold changes. The biomarker clusters dendrogram was cut based on similar patterns of fold changes for a subset of metabolites (see Results). Clustering was performed using the R packages ComplexHeatmap (41) and NbClust (42).

### 2.8. Participant phenotypic and demographic information

CAMP participant phenotypic and demographic information were based on physical and neurological examinations and behavioral testing performed by clinicians as well as a parental questionnaire. CAMP information from autistic and/or TYP children related to demographic information, diet, medications, behavioral assessments, or co-occurring conditions were selected for association analysis. The selected information was filtered to remove questions missing responses in more than 10% of autistic participants or that had an identical response in >98% of participants. The percent of ideal body weight (IBW) was based on the method of Traub and Johnson (43).

### 2.9. Association analyses

Association analysis of the biomarker values, biomarker defined subpopulations and the number of biomarker clusters to the CAMP demographic or phenotypic variables of autistic children was performed. To test for associations using biomarker values or the number of biomarker clusters, partial Spearman’s correlation coefficients rho (ρ) with age as a covariate were used when a demographic or phenotypic variable was continuous and a Kruskal-Wallis test was used when the metadata variable was categorical. Response wise Wilcoxon rank sum tests were used as *post hoc* tests for the Kruskal-Wallis tests. Association analysis between biomarker positive and negative populations and CAMP metadata was performed using Fisher exact tests for categorical variables and Welch t-tests for continuous metadata variables. *Post hoc* tests for Fisher exact tests of categorical metadata variables were performed by creating a dichotomous response variable for each response of the categorical variable followed by a response wise Fisher exact test. Effect sizes were reported using Cohen’s d for Welch t-tests, r statistic for Wilcoxon independent two sample tests, bias corrected Crammer’s V or odds ratios for Fisher’s Exact tests, and rank eta squared (η^2^) for Kruskal-Wallis tests. False discovery rate corrections (adj. *p*-value) were performed to control for multiple comparison testing (44). Partial correlations were calculated using the ppcor R package (45).

## 3. Results

### 3.1. CAMP study participant population

Plasma samples from 708 CAMP participants corresponding to 499 autistic and 209 typically developing children were utilized in this study. The autistic population was 2.5 months older than the TYP population (*p*-value <0.05, Table 1). The autistic population also had a higher proportion of males (79% versus 59%, Table 1). Asians and those who did not specify a race were overrepresented and Black or African Americans were underrepresented in the autistic population (Table 1). Other demographic factors found in Table 1 were balanced between the autistic and TYP populations. Parental interviews, medical records, and assessments by clinicians were used to evaluate co-occurring conditions associated with autism in the CAMP population as summarized in Supplementary Table 2.

**Table 1:** CAMP population.

Metric	Autistic	Typical
N	499	209
Age (months)*	35.1 ± 7.8	32.6 ± 8.7
BMI	16.7 ± 2.1	17 ± 2.5
IBW (%)	101.2 ± 12.8	103.2 ± 15.1
Male (%)*	79	59.3
Native American (%)	1	0
Asian (%)*	6	1.4
Black (%)*	6.8	19.1
Pacific Islander (%)	0.2	0
Race not specified (%)*	14.6	7.7
White (%)	71.3	71.8
MSEL scores		
Overall developmental quotient*	62.7 ± 17.3	101.7 ± 16.3
Expressive language*	28.1 ± 10.5	49.7 ± 9.5
Receptive language*	27 ± 11.4	50.5 ± 10
Fine motor*	28.4 ± 10.7	48.8 ± 10.9
Visual reception*	31.4 ± 13.6	54.1 ± 12.9
Diet and medication		
Medication (%)*	66	36
Preferred diet (%)*	62	19
Special diet (%)*	16	9
ADOS-2 CSS		
Comparison severity	7.1 ± 1.8	Not performed
Social affect	6.9 ± 1.7	Not performed
Restrictive repetitive behavior	7.8 ± 1.7	Not performed

*Indicates a comparison between the autistic and typical populations with a statistically significant difference (*p*-value < 0.05).

ADOS-2, Autism Diagnostic Observation Schedule-Second Edition; BMI, body mass index; IBW, percentage of ideal bodyweight; CSS, Calibrated Severity Score; MSEL, Mullen Scales of Early Learning.

### 3.2. Identification of metabolic biomarkers of autism-associated subpopulations

We quantified 54 plasma metabolites comprised of organic and amino acids, acylcarnitines, purines and microbiome-associated metabolites that have been of interest in autism research (15–17, 20, 26, 46, 47) (Supplementary Table 1). We also evaluated 1,275 unique ratios of these metabolites. Our aim was to test if a quantitative threshold of the concentration of a metabolite or the value of a ratio of metabolites could serve as a biomarker of an autism-associated subpopulation (Figure 1). Of the metabolites and ratios of metabolites that were examined, 153 autism-associated biomarkers were identified. The biomarkers included 4 individual metabolites and 149 ratios of metabolites (Supplementary Table 3). Each biomarker identified a subpopulation that contained between 4.5% and 11% of the CAMP study autistic population. The subpopulations were comprised of 90–100% autistic participants. One or more biomarkers identified 414 (83%) of the CAMP study autistic participants. The most frequent metabolites to appear as biomarker numerators or denominators were lactate, carnitine, pyruvate, leucine, glycine, octanoylcarnitine, citrate, 4-hydroxyproline, phenylalanine, and 2-ketoglutarate. The metabolites 3-aminoisobutyric acid, serotonin, inosine, 3-indoxyl sulfate, 4-ethylphenyl sulfate, CMPF, dodecanedioic acid, hydroxybutyrylcarnitine, indoleacetic acid, indolelactic acid and p-cresol sulfate were not identified as biomarkers.

### 3.3. Clusters of biomarkers

**Figure 2:**
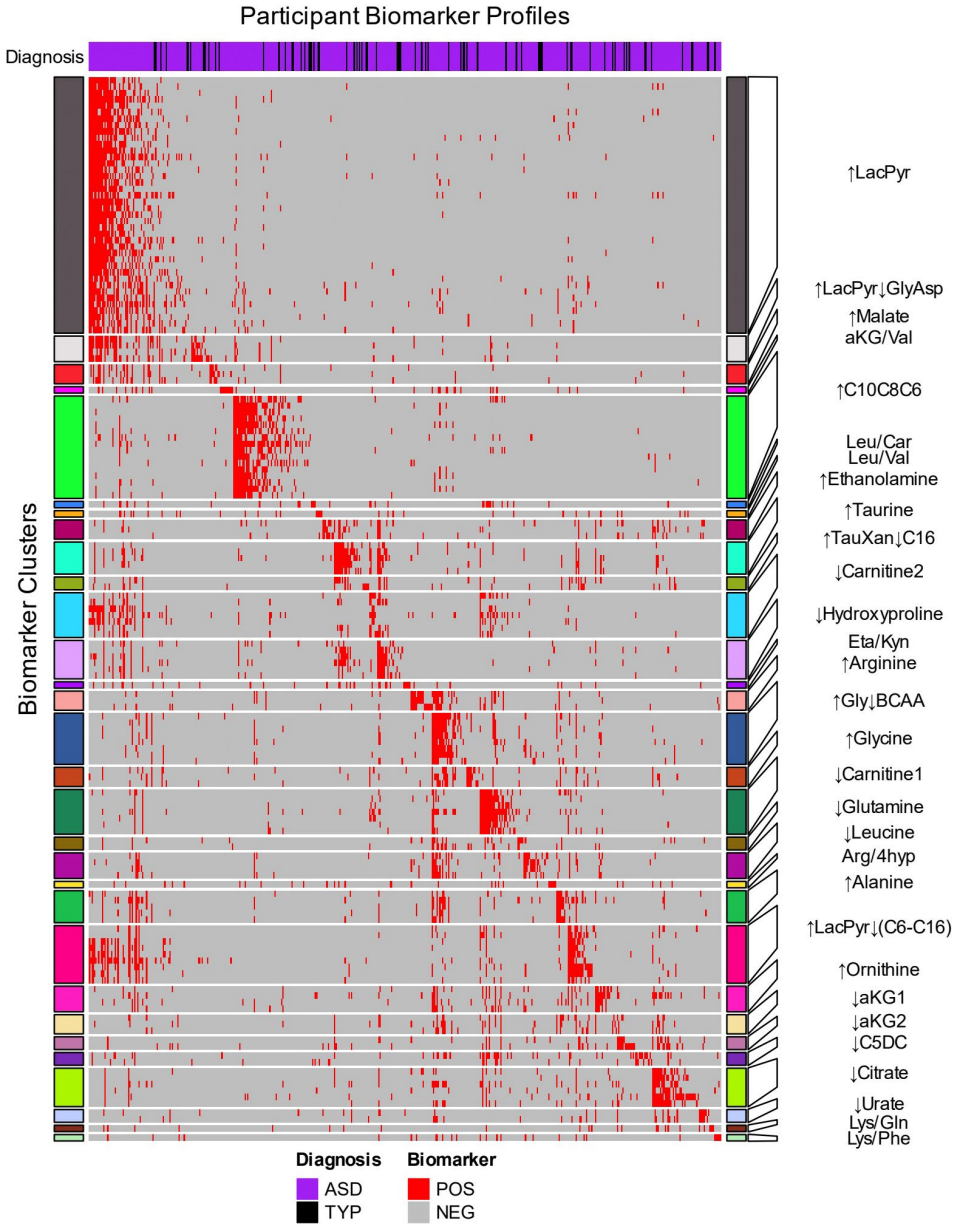
Hierarchical clustering of biomarkers based on the biomarker profiles of CAMP participants. The 153 biomarkers (rows) were clustered using the 414 CAMP autistic and 73 typically developing participants (columns) that were identified by at least one biomarker. The row names (biomarkers) are depicted in Supplementary Table 5. Red cells indicate when a biomarker identifies an individual within an autism-associated subpopulation (positive) and gray cells indicate when the biomarker does not identify an individual (negative). The heatmap rows are split into 30 clusters of related biomarkers (see Methods) that are designated by colored row blocks separated by white horizontal lines. The biomarker clusters were named using representative metabolite(s) that exhibited consistent increases (↑) or decreases (↓) in the biomarker positive relative to the biomarker negative participants. The column color bar at the top of the heatmap indicates the diagnosis: autism (purple) and TYP (black). The participants’ biomarker profiles (columns) were arranged by assigning each participant to a biomarker cluster in which their profile had the greatest number of biomarkers with a positive result.

An autistic participant is often identified by more than one of the 153 biomarkers (median 6 biomarkers, range 1–62, Figure 2, columns). The identification of an individual by multiple biomarkers suggested that the biomarkers may be identifying related metabolic processes (Supplementary Table 4). To explore this hypothesis and reduce complexity of the biomarkers, the biomarkers that often co-identify participants were grouped together using hierarchical clustering (Figure 2 rows). The 153 biomarkers formed 30 clusters; 23 contained 2 to 40 biomarkers and 7 clusters each consisted of 1 biomarker (Figure 2 rows, Supplementary Table 5). The biomarker clusters ↑Glycine, ↑Ornithine, ↑Gly↓BCAA, ↓Leucine, ↑LacPyr, ↑LacPyr↓GlyAsp, ↓aKG1, ↓Urate, and Arg/4hyp contain additional metabolites and ratios of metabolites that were not evaluated in our earlier work (16, 26). New biomarker clusters include those that contain the metabolites malate, citrate, xanthine, hypoxanthine, taurine, carnitine and the indicated acylcarnitines.

The autism-associated biomarkers can either be used by themselves or clustered with other autism-associated biomarkers to identify autism-associated metabolic phenotypes. The 30 biomarker clusters each identified subpopulations with a range of 5% to 28% of the CAMP autistic participants and were each comprised of 89% to 97% of autistic individuals (Table 2). As with individual biomarkers, participants are often identified by more than one biomarker cluster (median 3 biomarker clusters, range 1–19) with 52% of study participants identified by more than one biomarker cluster. The observation that different biomarker clusters often co-identify an individual (Supplementary Figure 3; Supplementary Table 6) suggests that the metabolic clusters are not independent of each other, i.e., there are alterations of metabolism that commonly occur together in the subpopulations.

**Table 2:** Biomarker cluster information.

Biomarker cluster	Number of biomarkers	% ASD	% PMP-ASD
↑LacPyr	40	27.6	90.1
↑LacPyr↓GlyAsp	4	13.3	95.7
↑Malate	3	10.1	96.2
aKG/Val	1	7.6	95.1
↑C10C8C6	16	19.3	90.5
Leu/Car	1	5.8	90.4
Leu/Val	1	6.6	91.3
↑Ethanolamine	3	13.3	93
↑Taurine	5	13	92.9
↑TauXan↓C16	2	10	94.2
↓Carnitine2	7	18.1	96.8
↓Hydroxyproline	6	12.8	88.9
Eta/Kyn	1	7.2	89.6
↑Arginine	3	11.2	91.8
↑Gly↓BCAA	8	16.2	93.1
↑Glycine	3	12	95.2
↓Carnitine1	7	15.7	95.1
↓Glutamine	2	8.2	95.3
↓Leucine	4	12.8	92.8
Arg/4hyp	1	6.7	94.2
↑Alanine	5	13	94.2
↑LacPyr↓(C6-C16)	9	18.3	93.8
↑Ornithine	4	18.2	95.8
↓aKG1	3	11.7	96.7
↓aKG2	2	11.7	93.5
↓C5DC	2	12.8	92.6
↓Citrate	6	19.3	92.3
↓Urate	2	7.8	92.9
Lys/Gln	1	5.6	94.2
Lys/Phe	1	5.7	92.7

%ASD, percentage of CAMP autistic participants (sensitivity), %PMP-ASD, percentage of the subpopulation that are autistic; Lac, lactate; Pyr, pyruvate, aKG, alpha-ketoglutarate; Xan, xanthine; Car, carnitine; BCAA, branched chain amino acid; C5DC, glutarylcarnitine; C6, hexanoylcarnitine; C8, octanoylcarnitine; C10, decanoylcarnitine; C16, palmitoylcarnitine. Eta, ethanolamine; Kyn, kynurenine; 4Hyp, 4-hydroxyproline.

### 3.4. Biomarker clusters exhibit patterns of metabolite fold changes that reveal autism-related metabolism

**Figure 3:**
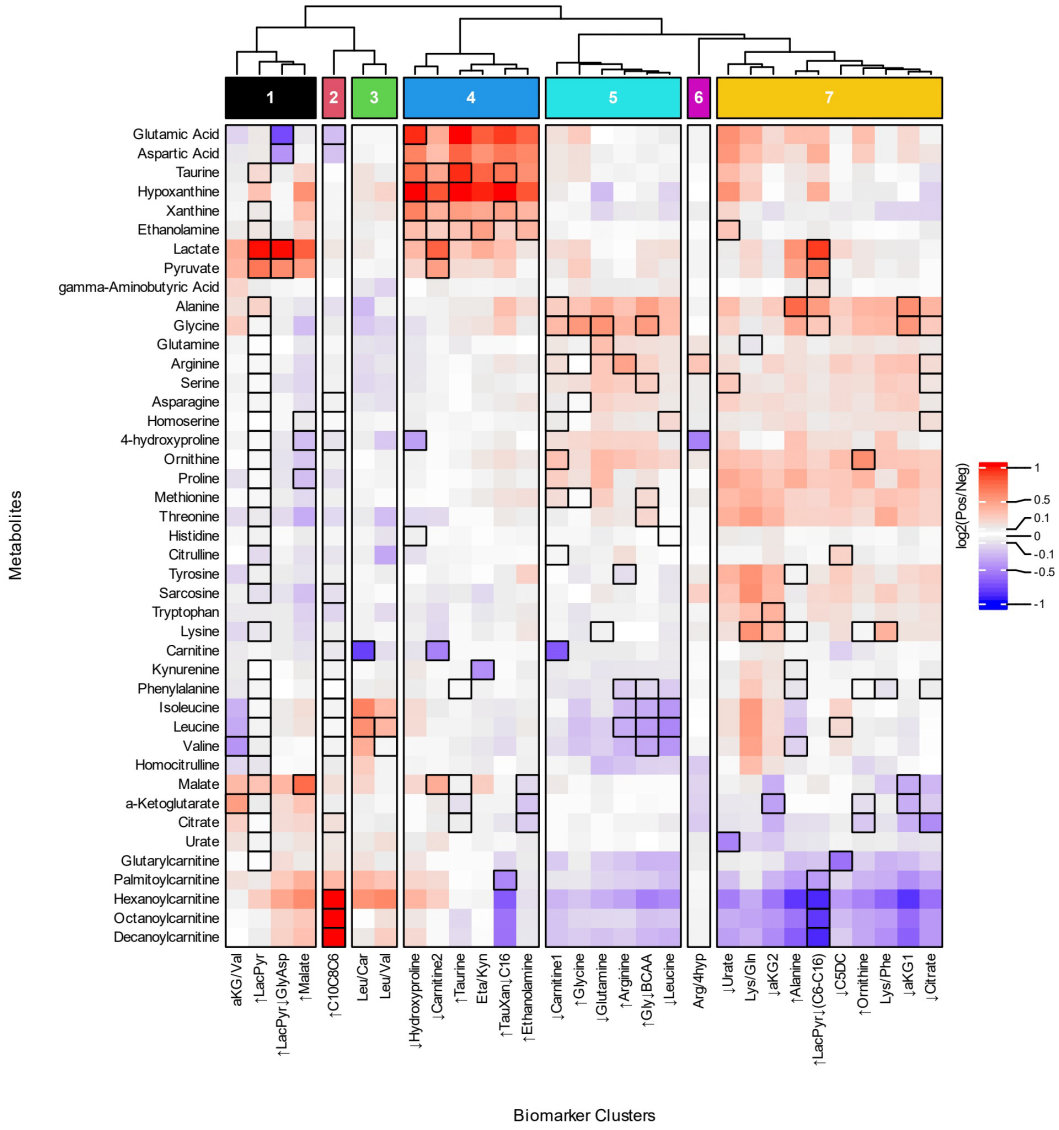
Heatmap of metabolite fold changes and grouping of the biomarker clusters. The metabolites (rows) selected for the heatmap occur as a numerator, denominator or individual metabolite of the biomarker clusters. The heatmap cells are fold changes (Supplementary Table 7) of the mean metabolite concentrations between the participants identified by a biomarker cluster (Pos) and those that were not (Neg). The dendrogram was cut based on similar patterns of fold changes for the acylcarnitines, branched chain amino acids, lactate, and pyruvate into seven groups of biomarker clusters, Groups 1–7, that are indicated at the top of the figure.

To investigate the relationship of biomarker clusters and metabolism, we compared the fold changes of metabolites between biomarker cluster-positive and negative participants. We evaluated the changes in metabolites to obtain a more precise understanding of the metabolic processes and pathways associated with biomarker clusters that could not be derived from ratios of metabolites. Metabolites with the greatest differences in concentrations between cluster positive and negative populations provide information about the influence of a metabolite in a biomarker cluster’s metabolic phenotype. Patterns of metabolite changes among the biomarker clusters can then be used to group biomarker clusters based on the similarity of fold changes across metabolites. We grouped the biomarker clusters fold changes and selected 7 groups of biomarker clusters based on acylcarnitines, branched chain amino acids, lactate, and pyruvate (Figure 3; Supplementary Table 7). By focusing on metabolites with the greatest changes and grouping the biomarker clusters, we were able to reduce the complexity of our analysis and obtain more meaningful biochemical interpretation of the biomarker clusters which are described below.

Four groups of biomarker clusters had different patterns of fold changes where increased acylcarnitines were evident among them (Figure 3). Group 1 consists of the ↑LacPyr, aKG/Val, ↑LacPyr↓GlyAsp, and ↑Malate clusters and is present in 35% of autistic participants. The group is characterized by increases in lactate, pyruvate, and malate, with varied increases in saturated medium and long chain acylcarnitines. A second pattern of fold changes is evident in the ↑C10C8C6 cluster of group 2 and is observed in 19% of the autistic participants. This cluster’s fold changes are characterized by strongly increased medium chain acylcarnitines, elevated palmitoylcarnitine and decreased glutamate and aspartate. The third pattern of fold changes with increased acylcarnitines consists of two clusters, Leu/Car and Leu/Val, is present in 11% of participants with autism and is characterized by increased levels of hexanoylcarnitine, palmitoylcarnitine and the branched chain amino acids (BCAAs) and decreased carnitine.

Two groups of biomarker clusters had a pattern of fold changes with decreased levels of acylcarnitines (Figure 3, groups 5 and 7). The clusters ↓Carnitine1, ↑Glycine, ↓Glutamine, ↑Arginine, ↑Gly↓BCAA, ↓Leucine, that together comprise 37% of the autistic participants, are characterized by decreased levels of the BCAAs, medium and long chain acylcarnitines and glutarylcarnitine, in combination with increased alanine, glycine, and ornithine. Another pattern of fold changes is present in 50% of autistic participants, shared by the ↓Urate, Lys/Gln, ↓aKG2, ↑Alanine, ↑LacPyr↓(C6-C16), ↓C5DC, ↑Ornithine, Lys/Phe, ↓aKG1, and ↓Citrate clusters, had decreased medium and long chain acylcarnitines, decreased glutarylcarnitine, variably decreased TCA cycle metabolites and variably increased alanine, glycine, threonine, methionine, proline and ornithine.

The biomarker clusters ↓Hydroxyproline, ↓Carnitine2, ↑Taurine, Eta/Kyn, ↑TauXan↓C16, and ↑Ethanolamine in group 4 (Figure 3, group 4) exhibit a pattern of fold changes occurring in 40% of the autistic participants. Increases of aspartate, glutamate, hypoxanthine, xanthine, taurine, ethanolamine, lactate and pyruvate are associated with this cluster. A pattern of fold changes noted in a single cluster, Arg/4Hyp (Figure 3, group 6), shows decreased 4-hydroxyproline and tricarboxylic acid cycle (TCA) intermediates and increased arginine. These clusters do not exhibit consistent changes in the acylcarnitines.

In terms of specific metabolites, decreased free carnitine is noted in three clusters that appear in different groups, ↓Carnitine1, ↓Carnitine2 and Leu/Car (Figure 3, groups 3–5). These biomarker clusters are observed in 26% of autistic participants. Each of these is associated with a distinctive metabolic profile. The first has decreased medium and long chain acylcarnitines and increased alanine, glycine, ornithine and proline. The second has increased lactate, pyruvate, malate and hypoxanthine and minimal changes of acylcarnitines. The third is characterized by increased BCAAs, hexanoylcarnitine and palmitoylcarnitine.

### 3.5. Biomarkers as a tool for screening metabolic differences associated with pediatric autism

The biomarkers use quantitative thresholds to identify potentially diagnostic subpopulations of the autistic participants that are comprised of at least 90% autistic individuals. The thresholds provide an opportunity for the biomarkers to be used as “tests” for biomarker levels that are associated with a likelihood of autism. The tests individually have high specificities (>98%) but low sensitivities (5–11%). The high specificity of these tests allows for a ‘stacking approach’ whereby a subset of the 153 biomarker-based tests can be combined into a battery of tests that increases overall test sensitivity for a likelihood of autism. Using a test optimization process to maintain a specificity of at least 90% (26), a subset of 42 biomarkers was selected that identified CAMP autistic participants with a sensitivity of 72% and specificity of 90% (Supplementary Table 8).

### 3.6. Associations of CAMP behavioral and phenotypic data with biomarker quantitative values

The levels of biomarkers corresponding to metabolite concentrations and ratios of metabolite values from autistic participants were evaluated for associations with behavioral test scores, demographic factors, co-occurring conditions, diets, and medications.

#### 3.6.1. Biomarker value associations with demographic factors and diet

The age of the participants was correlated (*ρ* = −0.26 to 0.15, adj. *p*-values <0.1) with 50 biomarkers primarily consisting of metabolite ratios containing lactate, pyruvate, a TCA intermediate, xanthine or hypoxanthine (Supplementary Table 9). BMI was correlated with Taurine/Ethanolamine (*ρ* = −0.15, adj. *p*-value = 0.02) and Alanine/2-Ketoglutarate (*ρ* = 0.15, adj. *p*-value = 0.04) (Supplementary Table 9). The carnitine associated biomarkers Glycine/Carnitine (*η*^2^ = 0.02, adj. *p*-value = 0.05) and Methionine/Carnitine (*η*^2^ = 0.03, adj. *p-*value = 0.03) were increased in children with preferred diet (food selectivity) (Supplementary Table 10). The levels of Arginine/Tyrosine (*η*^2^ = 0.03, adj. *p-*value = 0.03) and Lactate/Tyrosine (*η*^2^ = 0.02, adj. *p-*value = 0.05) were increased in participants on special diets (Supplementary Table 10). The ornithine containing biomarker Ornithine/Phenylalanine (*η*^2^ = 0.02, adj. *p*-value = 0.05) was increased in females compared to males (Supplementary Table 10); no other biomarker associations with sex were noted. The ratios of the metabolites Glycine/Arginine (*η*^2^ = 0.04, adj. *p*-value = 0.03) and Glycine/Methionine (*η*^2^ = 0.04, adj. *p*-value = 0.05) exhibited lower levels in Asians compared to White participants (Supplementary Table 10). Associations of biomarkers were not identified for those taking medications.

#### 3.6.2. Biomarker value associations with behavioral test scores

The biomarker Arginine/Tyrosine was associated with increased ADOS-2 CSS (*ρ* = 0.17, adj. *p*-value = 0.04) (Supplementary Table 9). Increased ADOS-2 RRB CSS scores were correlated with Ethanolamine/Kynurenine (*ρ* = 0.15, adj. *p*-value = 0.07) and Lactate/Threonine (*ρ* = 0.15, adj. *p*-value = 0.09). MSEL DQ values are negatively correlated with increased lactate (*ρ* = −0.12, adj. *p*-value = 0.07) and 8 lactate-containing ratios (*ρ* = −0.16–0.12, adj. *p*-value < 0.1) and these ratios are also negatively correlated with one or more scores in MSEL domains (Supplementary Table 9). Lactate, numerous lactate-containing-ratios, Ethanolamine/Citrate, and Alanine/Lysine levels are negatively correlated with MSEL receptive language, expressive language, and/or visual reception scores (Supplementary Table 9).

#### 3.6.3. Biomarker value associations with selected developmental milestones and co-occurring conditions

The biomarker ratio Taurine/Ethanolamine (*η*^2^ = 0.02, adj. *p*-value = 0.02) was increased with delayed attainment of walking (Supplementary Table 10). Delayed attainment of spoken phrases was associated (*η*^2^ = 0.03–0.04, adj. *p*-value < 0.1) with increased levels of lactate and metabolite ratios containing lactate (Supplementary Table 10). A prenatal history of maternal diabetes, a history of delays in attainment of first word spoken, rolling or sitting, of developmental regression or co-occurring conditions such as seizures, sleep problems, gastrointestinal problems, low muscle tone, macrocephaly or other physical abnormalities were not associated with biomarkers identified in this study at an adjusted *p-*value <0.1 (Supplementary Table 10).

### 3.7. Biomarker positive and negative population association analyses

The biomarker-positive autistic individuals identified by the individual biomarkers or biomarker clusters were tested for associations with CAMP autistic participants’ metadata. Participants identified by the biomarkers Methionine/Carnitine, Ornithine/Carnitine, Alanine/Tyrosine, Alanine/Phenylalanine or Ethanolamine/4-hydroxyproline were associated with lower DQ and MSEL subdomain scores (Supplementary Table 11). Individuals identified by Leucine and the biomarker clusters ↑LacPyr or ↑Malate were associated with a decreased MSEL Receptive Language Score, but not a decrease in DQ (Supplementary Table 11). Participants identified by Taurine/Malate were associated with lower BMIs (Supplementary Table 11). Other metadata were not significantly associated with participants identified by an individual biomarker or a cluster of biomarkers at a significance threshold of an adjusted *p*-value of 0.1 (Supplementary Table 12).

### 3.8. The number of biomarker clusters that identify a participant are associated with increased severity of autism and developmental delays

**Figure 4:**
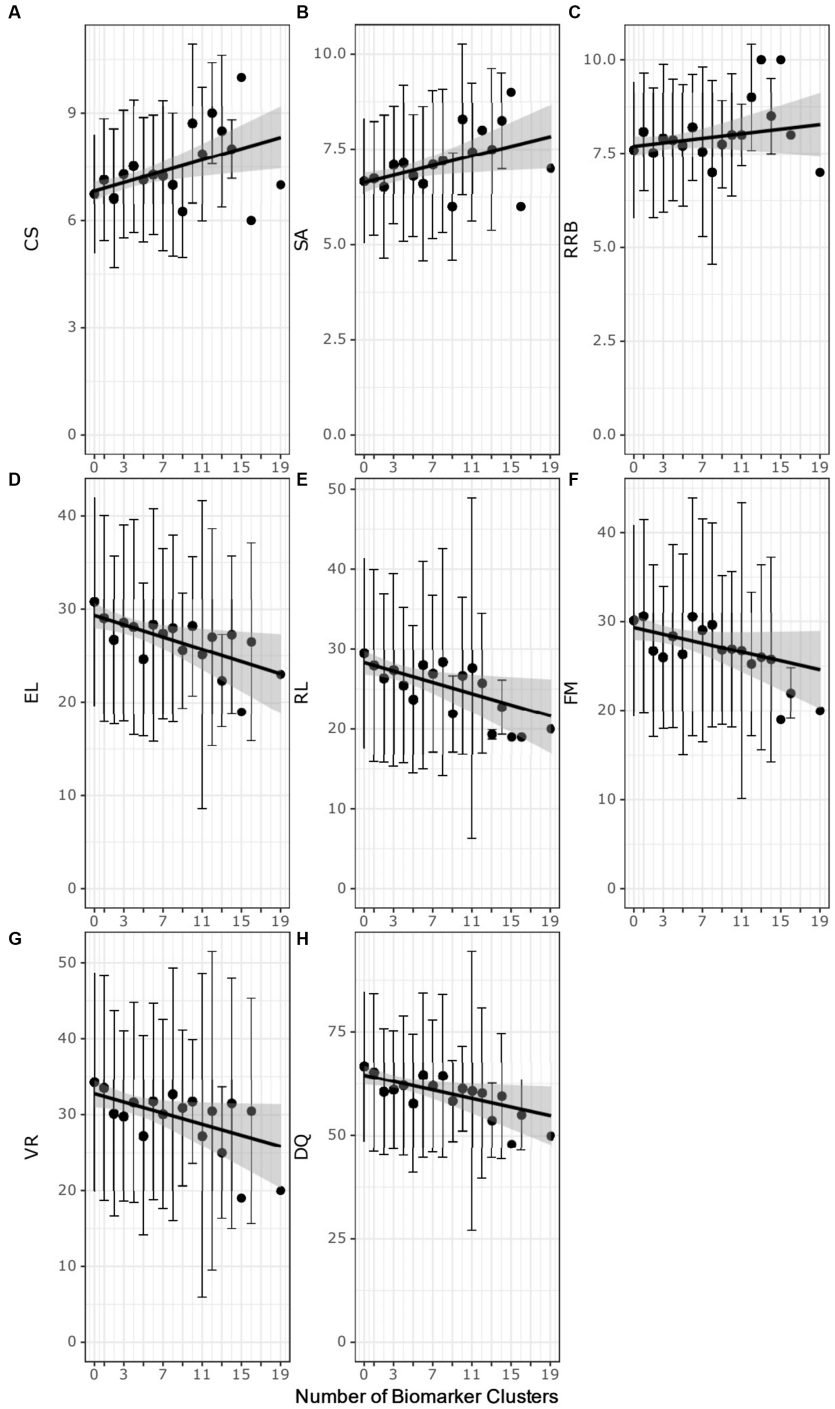
Scatter plots of the number of biomarker clusters in an individual’s biomarker profile and the ADOS-2 test scores **(A–C)** or the MSEL test scores **(D–H)** test scores. Each point represents the average test score at biomarker cluster number. The error bars correspond to ± the standard deviation. Black regression lines are fit using simple linear regression. The gray highlighted region is the 95% confidence interval of the regression model. CSS, composite severity score; SA, social affect calibrated severity score; RRB, restrictive repetitive behavior calibrated severity score; DQ, developmental quotient; RL Receptive language T-score; EL, expressive language T-score; VR, visual reception T-score; FM, fine motor T-score.

The autistic participants were evaluated for associations with autism severity based on the ADOS-2 test scores (27) and the number of biomarker clusters that identify an autistic participant (Figures 4A–C; Supplementary Table 13). Increased ADOS-2 CSS, indicating more severe autism characteristics, was positively associated with the number of biomarker clusters (*ρ* = 0.14, adj. *p*-value = 0.016). The number of biomarker clusters was also correlated (*ρ* = 0.12, adj. *p*-value = 0.05) with more severe SA CSS. The number of clusters was not associated with RRB CSS (*ρ* = 0.05, adjusted *p*-value = 0.32). That the number of clusters have an association with SA CSS and lack of association with RBB CSS suggests that the increased autism severity associated with increased number of clusters is related to deficits in social communication.

The autistic participants with increasing numbers of biomarker clusters exhibited more severe developmental delays as measured by lower MSEL test scores (Figures 4D–H; Supplementary Table 13). The DQ decreased with increasing numbers of clusters (ρ = − 0.13, adj. *p*-value = 0.008). Decreased DQ was associated with a negative correlation of expressive (*ρ* = −0.14, adj. *p*-value = 0.005) and receptive language (*ρ* = −0.14, adj. *p*-value = 0.005), fine motor (*ρ* = −0.13, adj. *p*-value = 0.013) and visual reception (*ρ* = − 0.12, adj. *p*-value = 0.016) with increasing numbers of clusters.

The number of biomarker clusters was not associated (Kruskal-Wallis test adjusted *p*-value > 0.1 or Spearman partial correlation test adjusted *p*-value>0.1) with age, race, BMI, diet, medications, sleep problems, gastrointestinal issues, food selectivity, or delayed attainment of early language or motor milestones (Supplementary Table 14). The lack of association with these factors raises the interesting possibility that an increased number of biomarker clusters is primarily associated with the severity of autism and reduced developmental quotients in the CAMP autistic population.

## 4. Discussion

The goal of this study was to identify metabolic biomarkers associated with subpopulations of autistic individuals that are largely absent in typically developing participants. Using quantitative measurements of 54 metabolites and their ratios in a carefully selected cohort of 499 autistic and 209 typically developing controls that are ages 18–48 months, we identified 153 autism-associated biomarkers. Clustering of the biomarkers based on similarities of the autistic participant’s biomarker profiles revealed 30 clusters of biomarkers. These biomarker clusters, in turn, formed 7 groups based on fold changes of free carnitine, selected acylcarnitines, the branched chain amino acids, lactate, and pyruvate and reveal patterns of metabolic dysregulation in autism. In addition, an optimized subset of the biomarkers identified autistic participants in the CAMP study with 72% sensitivity and 90% specificity. It is important to note that the ASD-associated phenotypes identified in this study were determined in a population of individuals that are presumed to have idiopathic autism. The latter corresponds, by far, to the categorical designation of most people with ASD at the current time.

Central to our identification of metabolic subpopulations of autism is the use of quantitative thresholds of ratios of metabolites that can be used as biomarkers. This type of metabolite ratio analysis can increase diagnostic efficacy by detecting changes not apparent when using an analysis of individual metabolites thereby providing information on biological processes that may not be discerned when studying only metabolite levels (34). Metabolite ratios have been successfully used in newborn screening for inherited metabolic disorders such as phenylketonuria, maple syrup urine disease, and certain disorders of organic acid or of mitochondrial fatty acid metabolism (35, 36) as well as in identifying metabolic underpinnings of more complex, multifactorial biological processes (37, 38). This study is an application of this approach for metabolic phenotyping and biomarker discovery in autism.

**Figure 5:**
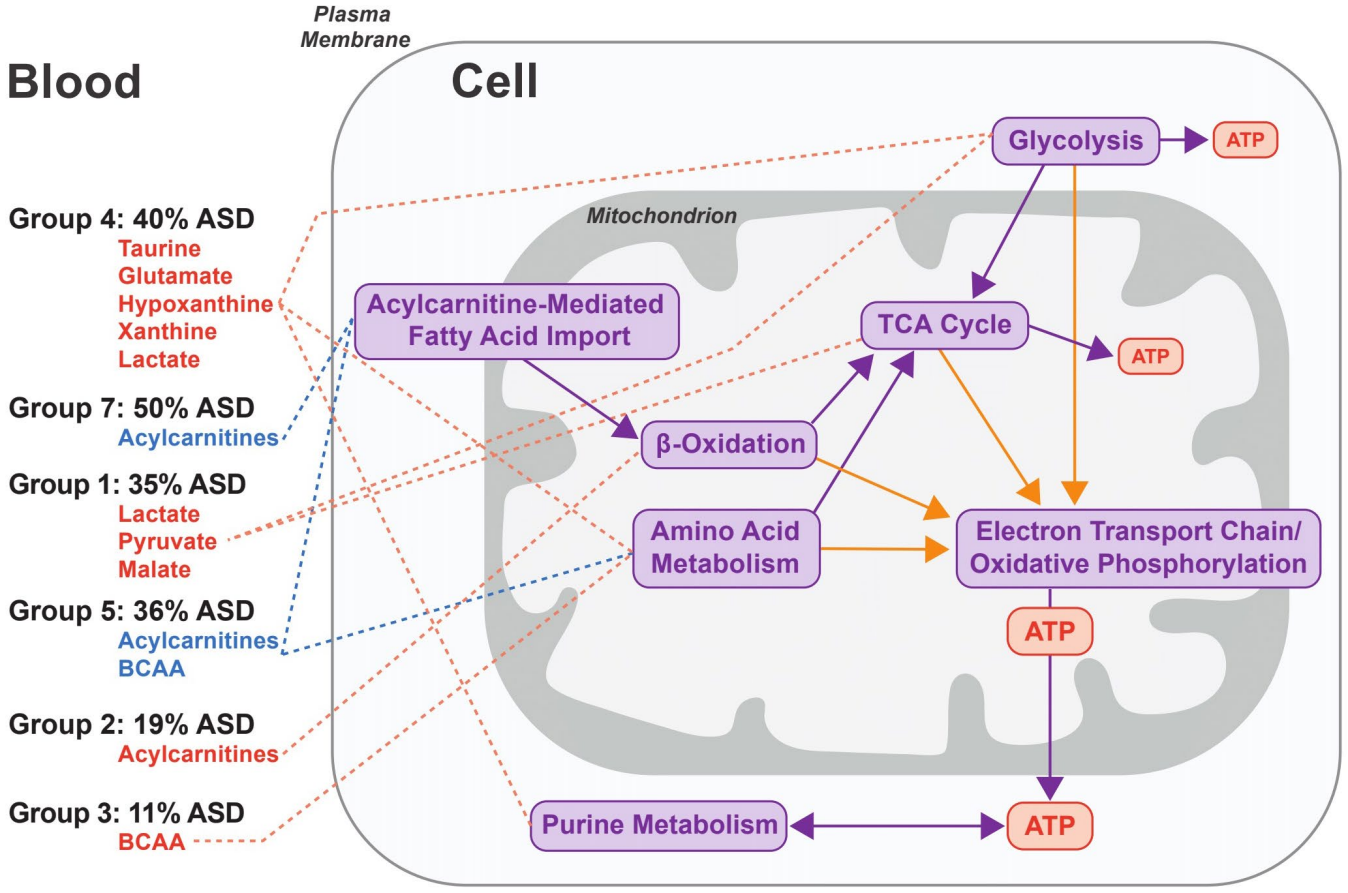
Model of the metabolic processes associated with groups of biomarker clusters. The figure represents an idealized cell and the metabolic processes implicated in autism pathobiology that are related to the patterns of metabolite fold changes in the 6 groups of biomarker clusters comprised of more than one biomarker (see Figure 3 groups 1–5, and 7). Metabolites that are elevated are depicted in red and decreased metabolites are indicated in blue for each of the cluster groups. For each group, the percentage of autistic CAMP children positive for at least one cluster in a group is indicated. Metabolite pathways (purple arrows) and redox processes (orange arrows) that relate to energy generation via mitochondrial oxidative phosphorylation are shown on the right side of the figure. Decreases of carnitine in 3 biomarkers clusters (↓Carnitine1, ↓Carnitine2, and Leu/Car) contained within groups 3–5 indicate that low carnitine may occur with different metabolic states in autism. Group 4 exhibits changes associated with increased glycolysis or glycogenolysis from muscular activity and the increased purines could arise from nucleoside metabolism associated with muscular activity. Lactate and pyruvate levels can be increased in diverse contexts. They are often increased in instances of altered function of oxidative phosphorylation or of the TCA cycle that are consistent with changes in Group 1 and some clusters in Group 7 that have increased lactate and pyruvate. Increased plasma acylcarnitines in group 2 are indicative of altered cellular bioenergetics that occurs when the capacity of complete mitochondrial fatty acid beta-oxidation is exceeded. Decreased acylcarnitines in the biomarker clusters associated with groups 5 and 7 likely relate to reduced fatty acids from dietary intake or derived from lipolysis or due to decreased fatty acid entry into the mitochondria via the long chain fatty acid uptake/mitochondrial CPT1/CACT/CPT2-mediated transport process. Decreased BCAAs in group 5, together with decreased acylcarnitines, may reflect a relative hyperinsulinemic state.

The fold changes of metabolites used to group biomarker clusters provide insights into the metabolic processes related to autism pathobiology (Figure 3). Overall, the fold change data indicate heterogenous patterns of altered cellular bioenergetics, especially mitochondrial dysregulation, associated with a majority of the CAMP autistic participants (Figure 5). Disturbances of mitochondrial biology, primarily mitochondrial bioenergetics, have a long history as a correlate of, and contributing factor to, the pathobiology of autism (48, 49). This connection to autism has been supported by a set of rare primary mitochondrial and nuclear genetic disorders that sometimes have autism as a clinical manifestation (48, 49). In addition, a subset of autistic people have strong biochemical evidence of mitochondrial dysregulation, especially of mitochondrial bioenergetics, although the percentages vary widely across studies depending on the autistic population studied and the biomarkers used (50). Brain neuroimaging studies and enzymatic, transcriptomic and proteomic analyses of the autistic brain further support forms of mitochondrial dysfunction in the central nervous system (51–57). Metabolic studies of cerebral organoids derived from autistic individuals also show decreased ATP production and mitochondrial respiratory chain activity (58). Because the brain has the highest mitochondrial energy demand of any organ, even subtle changes in mitochondrial energy production preferentially can affect brain function. For these reasons, and because of the complex nature of mitochondrial genetics, it has been proposed that many people with common neuropsychiatric disorders, including autism, have dysregulation of mitochondrial energy metabolism (59). The results of this study provide additional support for a role of altered regulation of mitochondrial energy metabolism in the pathobiology of autism (Figure 5).

The composition of biomarkers within the clusters suggests that multiple aspects of cellular bioenergetics are driving the clustering of biomarkers (Figure 5). The biomarker clusters with increased lactate, pyruvate, alanine, and sometimes tricarboxylic acid cycle (TCA) intermediates (Figure 5, group 1), suggest dysfunction of oxidative phosphorylation or the TCA cycle. However, the basis for the increased lactate and pyruvate, in some instances, may relate to non-mitochondrial processes such as glycolysis as is suggested by the absence of increased alanine and TCA intermediates noted in some other biomarker clusters (Figure 5, group 4); it also is possible that individuals in this group were substantially more agitated during their blood draws.

Different patterns of plasma medium and long chain acylcarnitine alterations usually occurred with other biomarkers of mitochondrial energy metabolism (Figure 5, groups 2, 5, and 7). Abnormalities of subsets of plasma acylcarnitines have been previously observed in autism (20, 60–63). Neither high (Figure 5, group 2) nor low levels (Figure 5, groups 5 and 7) of plasma medium and long chain acylcarnitines noted in our work correlate with described genetic disorders of mitochondrial fatty acid or organic acid metabolism in humans (64). Increased plasma medium and long chain acylcarnitines (Figure 5, Group 2) can otherwise result from different processes such as increased physical activity or extended fasting in which the efflux of acylcarnitines exceeds the capacity for complete oxidation of the fatty acids (65, 66). Reduced levels of plasma medium and long chain acylcarnitines, seen in other autistic participants (Figure 5, Groups 5 and 7) can result from a reduced dietary intake of fatty acids or from an inhibition of long chain fatty acid entry into the mitochondria upon increased lactate and downstream inhibition of carnitine palmitoyltransferase 1 (CPT1) activity (67).

As illustrated in Figures 2, 3, decreased free carnitine was noted in a subset of CAMP autistic participants. Carnitine is a central metabolite in the mitochondrial carnitine cycle/fatty acid oxidation pathway, in the transfer to mitochondria of the end products of peroxisomal fatty acid oxidation, and in Coenzyme A homeostasis. Individuals with low carnitine may have reduced carnitine synthesis; others may have abnormalities of carnitine intake, transport or loss. Previous studies indicate that both low and high levels of free carnitine have been associated with autism (61, 62, 68–70). Most of the data regarding plasma free carnitine and autism, however, relate to low levels of free carnitine. An association of hypocarnitinemia with a common, X-linked variant of reduced carnitine synthesis has been described (69). Subsequent efforts using supplementation with carnitine to treat people with autism and carnitine deficiency has had some success (47, 71, 72). Based on these findings, it has been proposed that brain carnitine deficiency causes male-biased, nonsyndromic autism (73). A significant observation in our data is that people with autism and low plasma free carnitine are metabolically diverse and there should therefore be no *a priori* expectation that all individuals with autism and low free carnitine will have similar autism-related pathobiology or identical clinical responses to carnitine supplementation.

Increased plasma xanthine or hypoxanthine were present in the biomarker profiles of 19% of CAMP autistic participants (Figure 5, Group 4). While this appears to be a novel finding (23, 24), abnormal levels of urinary hypoxanthine have been reported in autism and abnormal plasma and urine levels of other purines are noted in some rare monogenic metabolic disorders that are sometimes associated with autism (21, 74, 75). Clinical correlates of increased plasma hypoxanthine include increased muscle activity and hypoxia (76–79). Increased plasma xanthine and hypoxanthine can also occur consequent to disorders of mitochondrial oxidative phosphorylation or of glycogen metabolism (77). In most instances, increased plasma hypoxanthine is an indicator of cellular ATP consumption or deficit (80).

Changes in the levels of biomarkers were associated with behavioral test scores, demographic features, and several co-occurring conditions of autistic children. Increased levels of lactate, lactate-containing ratios, and Ethanolamine/Citrate are associated with both increased autism severity and decreased MSEL scores. Increased levels of metabolites associated with cellular bioenergetics have been associated with poorer cognitive and adaptive MSEL scores (81). Delays in attainment of some important developmental milestones including delays in speech and in walking were associated with increased lactate and lactate containing ratios and with increased levels of taurine/ethanolamine, respectively. Other co-occurring conditions evaluated in this study were not associated with biomarker levels. The age of participants was associated with biomarker levels; associations of biomarker levels with other demographic factors including BMI, diet, sex or race were minimal. The association of only a single biomarker with the sex of the participants was less than expected since males are overrepresented in ASD and sex specific genetic associations are reported (82).

We observed an association between the number of biomarker clusters autistic participants exhibited and the severity of their autism and developmental delays. As the number of clusters increased, there was also an increase in the composite ADOS-2 CSS as well as the SA CSS subscore but not the RBB CSS subscore. The developmental quotients as well as expressive and receptive language, fine motor, and visual reception subscales decreased with autistic individuals having increasing numbers of clusters. Increased severity of autism has been associated with increased plasma levels of energy-related metabolites and increased social deficits have been associated with increased levels of plasma lactate and glutamine (81, 83, 84). These results suggest that individuals with certain metabolic states may experience more severe autism, especially as relates to its social manifestations as well as developmental delays.

There are several limitations that impact the interpretation of the results. The study did not include neurodevelopmental disorders other than autism limiting the understanding of the specificity of biomarkers and metabolic states. The young age of the children in this study likely limited diagnoses of common co-occurring conditions in autism, thereby limiting our understanding of how biomarkers might relate to conditions such as anxiety and attention-deficit/hyperactivity disorder (ADHD). We requested information about special diets but some relevant dietary information may not have been obtained. Full laboratory and genomic information were not available for study participants, thereby limiting other clinical correlations and additional understanding of underlying pathophysiology. The metabolic observations lack longitudinal measurements in the study participants which would be required to evaluate the stability of the biomarkers over time.

This study reveals numerous quantitative biomarkers that identify metabolic subpopulations of autistic individuals. These biomarkers have low sensitivities and high specificities that are individually of limited diagnostic value but, in aggregate, have associations with autism-related behaviors and metabolism. These biomarkers can be leveraged as an adjunctive tool for early screening of children considered likely to attain a diagnosis of autism (26). Biomarker profiling in this way raises the prospect of focusing on the pathophysiology of different metabolic subtypes of autism as well as supporting decisions in therapeutic interventions and clinical trial management (85, 86). The biochemical processes associated with biomarkers may reflect an adaptation to compensate for a metabolic defect, such as the apparent increase in glycolytic end products when some mitochondrial processes are compromised. Further study is required to interpret these observations. Even at this stage, where the understanding of the pathobiology of each of the biomarker clusters is incomplete, treatment of some autistic patients with low-risk interventions may benefit individuals with diverse forms of “mitochondrial autism.” For example, individuals with low plasma free carnitine can potentially benefit from carnitine supplementation. Those with branched chain amino acid deficiencies may benefit from BCAA supplementation. There are several rare monogenic disorders associated with autism whose metabolic phenotype includes low levels of the plasma BCAAs for which supplementation with BCAAs has, in some instances, provided various clinical, including developmental or behavioral, benefits (87–92). There may be benefit from carnitine or BCAA supplementation for some of the children with low carnitine or low BCAA-associated phenotypes noted in this study, although this requires future investigation. A combined metabolic analysis and targeted treatment approach such as that suggested by this study has the potential to address the specific biology of a child’s neurodevelopmental condition thereby resulting in more effective treatment and better outcomes.

## Data availability statement

The original contributions presented in the study are included in the article/Supplementary material, further inquiries can be directed to the corresponding author.

## Ethics statement

The studies involving humans were approved by the Western Institutional Review Board and the IRBs of all 8 participating clinical centers. The studies were conducted in accordance with the local legislation and institutional requirements. Written informed consent for participation in this study was provided by the participants’ legal guardians/next of kin.

## Author contributions

AS conducted bioinformatic analyses related to biomarker, clustering, and association analyses. AS, RB, and MN performed research for the manuscript. AS, ED, DA, RB, and MN wrote the manuscript. AS, ED, DN, DA, RB, and MN edited the manuscript. AS, RB, ED, and DA were involved in the design and execution of the Children’s Autism Metabolome Project. All authors contributed to the article and approved the submitted version.
