# Supplementary material for: Metabolomic biomarkers in autism: identification of complex dysregulations of cellular bioenergetics
Source: Front Psychiatry. 2023 Oct 2;14:1249578. doi: 10.3389/fpsyt.2023.1249578 (PMC10622772; doi:10.3389/fpsyt.2023.1249578)
Supplement: Supplementary file 16 [file Data_Sheet_1.docx]

Supplementary Material

**Metabolomic biomarkers in autism: identification of complex dysregulations of cellular bioenergetics**

Alan M. Smith, Elizabeth L. R. Donley, Denise M. Ney, David G. Amaral, Robert E. Burrier and Marvin R. Natowicz*

*** Correspondence:** Marvin R. Natowicz: [natowim@ccf.org](mailto:natowim@ccf.org)

**Supporting Methods**

**Chemicals**

Optima and high-performance liquid chromatography (HPLC)-grade reagents (water, acetonitrile (ACN), methanol (MeOH), isopropanol, acetic acid, formic acid) were purchased from Fisher Scientific. Ammonium acetate was purchased from Sigma-Aldrich. The AccQTag Ultra derivatization kit was purchased from Waters. Chemical reference standards and isotopically labeled internal standards and vendors are listed in **Supplemental Table 1**.

**Triple Quadrupole liquid chromatography tandem mass spectrometry (LC-MS/MS) Method for Quantitative Analysis of Biologic Amines**

Protein precipitation with cold methanol was employed for all plasma, calibration standards (CAL) and quality control (QC) samples. Samples were derivatized as described previously (1) using the AccQTag Ultra kit (Waters). In brief, samples were thawed at room temperature, and 50 µl sample was prepared by adding 25 µl internal standard solution and 150 µl methanol (-20 ˚C) to precipitate plasma proteins. Samples were vortex-mixed for 5 min and spun at 18,500 x g for 5 min at 4 ˚C. Derivatization of sample extracts were carried out by transferring 10 µl of the supernatant onto a 96 well plate containing 70 µl of AccQTag Ultra Borate Buffer, followed by an addition of 20 µl of AccQTag Ultra Derivatization Reagent. Samples were briefly mixed and heated to 55 ˚C for 10 minutes then transferred to the autosampler (4 ˚C) for injection. Analysis was performed using 2 µl derivatized sample on an Agilent 1290 ultra-high-performance liquid chromatography system (UHPLC) coupled to an Agilent G6490 Triple Quadrupole Mass Spectrometer (Agilent Technologies Santa Clara, CA) run in dynamic Multiple-Reaction-Monitoring (dMRM) mode. Analyte separation was achieved on an Acquity UPLC HSS T3, 1.8µm, 2.1 x 150mm (Waters) column using water and ACN both with 0.1% formic acid as mobile phases A and B, respectively. The chromatographic gradient is shown in **Supplemental Table 15A**. MS detection was carried out using electrospray ionization in positive ion mode. Information about the detection of analytes is shown in **Supplemental Table 1**. Agilent MassHunter Quantitative Analysis software (version B.06.00) was used to quantify analytes based on area-under-the-response-curve. Stable isotope labeled internal standards were used for each analyte to account for variations in the matrix. Samples with analytes below the lowest calibration level standard were reported as 0.00 concentration. Samples with analytes above the highest calibration level standard were reanalyzed at an appropriate dilution using water:methanol (1:1).

**Triple Quadrupole LC-MS/MS Method for Quantitative Analysis of Unpolar Microbiome Metabolites**

Protein precipitation with cold methanol (-20 ˚C) was employed for all plasma, CAL and QC samples. Samples were thawed on ice, and 50 µl sample was used for the analysis. A 25 µl internal standard mix aliquot was added to the sample, and proteins were precipitated by addition of 150 µl MeOH (20 ˚C). Samples were vortex-mixed for 5 min followed by centrifugation at 18,500 x g for 5 min at 4 ˚C. A 100 ul aliquot of the supernatant was transferred to a 96well plate for injection (1-2 µl). Multiple reaction monitoring (MRM) analysis was performed on a liquid chromatography (LC) mass spectrometry (MS) system consisting of an Agilent 1290 ultra-high performance liquid chromatography system (UHPLC) coupled to an Agilent G6490 Triple Quadrupole Mass Spectrometer (Agilent Technologies Santa Clara, CA). Agilent MassHunter Quantitative Analysis software (version B.06.00) was used for the quantitative LC-MS data analysis. Chromatographic separation was performed using an Acquity UPLC BEH C18, 1.8µm, 2.1 x 100mm (Waters). Column temperature was maintained at 30 ˚C. The mobile phase was composed of A) 0.1% acetic acid in 5mM ammonium acetate and B) 0.1% acetic acid in acetonitrile. The details for the stepped gradient elution are shown in **Supplemental Table 15B**. The six-minute chromatographic gradient ran at a flow rate of 0.3 ml / min, and MS detection was carried out using electrospray ionization in both positive and negative ion modes. Information about the detection of analytes is shown in **Supplemental Table 1**. To account for matrix effects, stable isotope labeled (SIL) internal standards were used for each analyte. Samples with analytes below the lowest calibration level standard were reported as 0.00 concentration. Samples with analytes above the highest calibration level standard were re-analyzed at an appropriate dilution using water:methanol (50:50).

**Triple Quadrupole LC-MS/MS Method for Quantitative Analysis of Plasma Organic Acid Metabolites**

Protein precipitation with cold methanol -20 ˚C was used for all plasma, CAL and QC samples. Samples were thawed on ice, and 50 µl sample was used for the analysis. A 25 µl internal standard mix aliquot was added to the sample and proteins were precipitated by addition of 150µl MeOH (-20 ˚C) and vortex-mixed for 5 min followed by centrifugation at 18,500 x g for 5 minutes at 4 ˚C. An aliquot of the supernatant (100 µl) was transferred to a 96well plate, and 1-2 µl was injected onto a Acquity UPLC HSS T3, 1.8μm, 2.1 x 150mm (Waters). Multiple reaction monitoring (MRM) analysis was performed on a liquid chromatography (LC) mass spectrometry (MS) system consisting of an Agilent 1290 ultra-high performance liquid chromatography system (UHPLC) coupled to an Agilent G6490 Triple Quadrupole Mass Spectrometer (Agilent Technologies Santa Clara, CA). Mobile phase A was 0.2% formic acid in water and mobile phase B was 0.2% formic acid in acetonitrile. A stepped gradient elution was performed as shown in **Supplemental Table 15C.** of 0.35 ml / min. The eight-minute chromatographic gradient ran at a flow rate of 0.35 ml / min with the column kept at 30 ˚C. MS detection was carried out using electrospray ionization in both positive and negative ion modes. Information about the detection of analytes is shown in **Supplemental Table 1**. To mitigate matrix effects, stable isotope labeled internal standards were used for each analyte. Agilent MassHunter Quantitative Analysis software (version B.06.00) was used to quantify analytes based on area-under-the-response-curve. Samples with analytes below the lowest calibration level standard were reported as 0.00 concentration. Samples with analytes above the highest calibration level standard were re-analyzed at an appropriate dilution using water:methanol (50:50).

**Supplementary Figures**

**Supplementary Figure 1**. CONSORT flow diagram of the CAMP case-control phenotyping and biomarker study. Abbreviations: ASD, autism spectrum disorder; DD, developmental delay; TYP, typically developing; LC-MS, liquid chromatograph mass spectrometry.


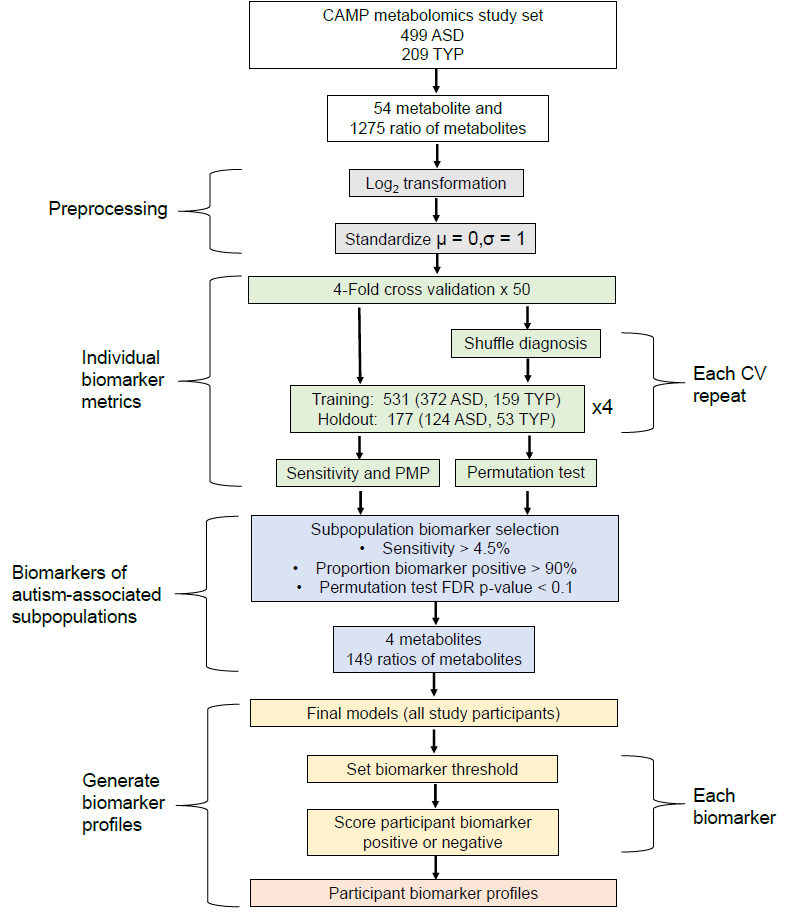


**Supplementary Figure 2**. Process used to identify biomarkers and generate participant biomarker profiles. Abbreviations: PMP, proportion biomarker positive; FDR, false discovery rate; CV, cross validation.


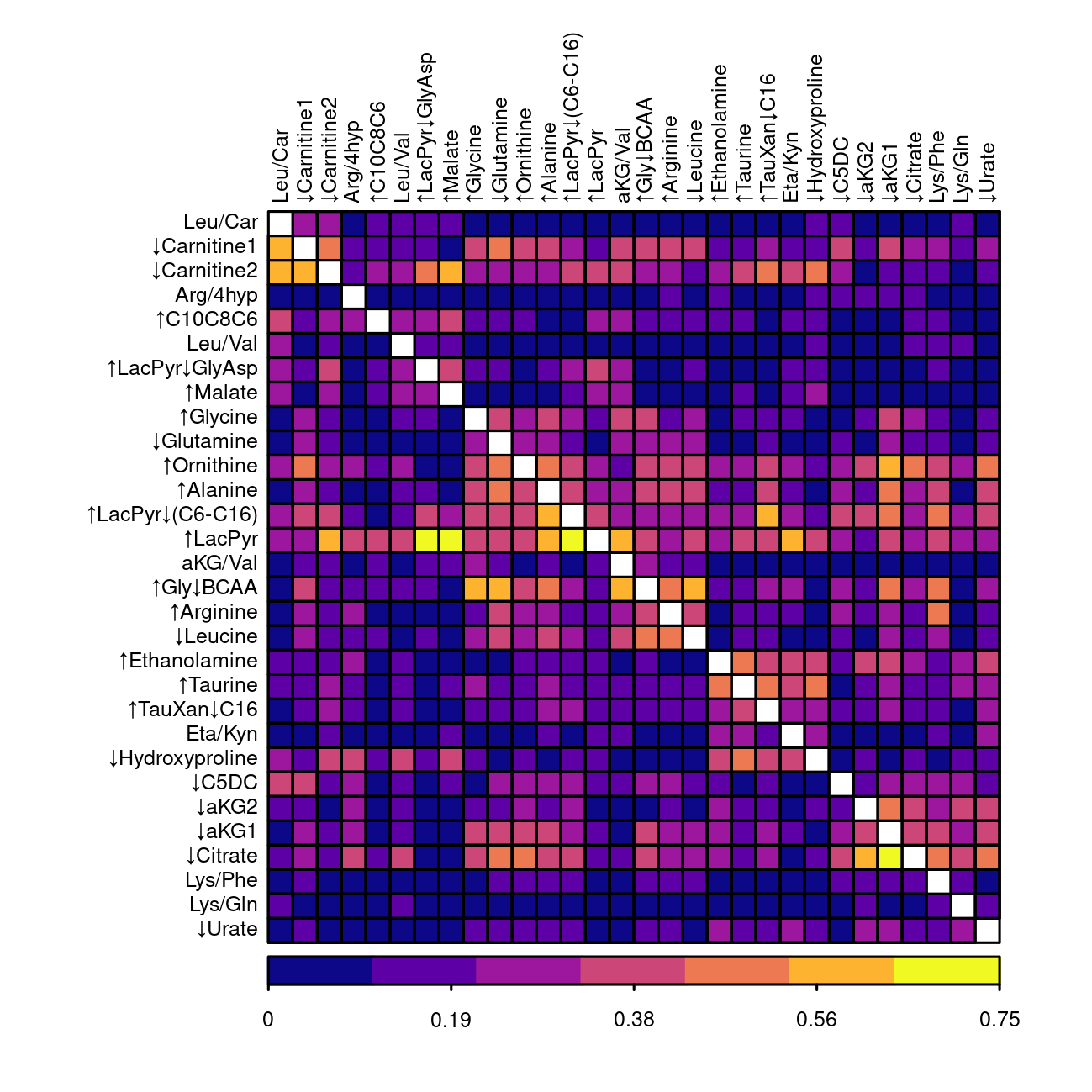


Supplementary Figure 3. Heatmap of the co-identification of participants by the biomarker clusters. The co-occurrence is presented as the conditional probability p(Column ∩ Row/Column) of participants being identified by a biomarker cluster in row given a being identified by a biomarker cluster in the column. The analysis provides a visualization for how often biomarker clusters co-identify a participant. The color scale indicates the conditional probability.

**Supplementary Tables**

The supplementary tables are persent as Excel files.

The table descriptions are below.

Supplementary Table 1. Metabolites, Chemical Reference Compounds, and Ions Measured by Mass Spectrometry Methods.

The ratio column indicates if the metabolite was used in ratios of metabolites (Yes) or was too sparsely measured for use in metabolite ratios (No). Abbreviations: End, endogenous metabolite; ISTD, spiked-in internal standard. CE, Collision Energy; CV, Capillary Voltage (Agilent), Cone Voltage (Waters); LC-MS/MS, liquid chromatography tandem mass spectrometry; CIL, Cambridge Isotope Laboratories; TRC, Toronto Research Chemicals; CDN, CDN Isotopes; SA, Sigma-Aldrich.

Supplementary Table 2. CAMP Population Co-occurring Conditions

Variable, CAMP phenotypic and behavioral data; Description, description of the CAMP phenotypic and behavioral data; OddsRatio, conditional Maximum Likelihood Estimate (MLE) of the odds ratio for 2×2 cases; ; p, Fisher Exact Test p-value; p.adj, false discovery rate (FDR) adjusted Fisher Exact Test p-value; Response, dichotomized response used for Fisher Exact Test post hoc, analysis; ASD, number of ASD participants with response; TYP, number of ASD participants with response; propASD, proportion of the total number of ASD participants with response; propTYP, proportion of the total number of TYP participants with response; OddsRatio.post, conditional Maximum Likelihood Estimate (MLE) of the odds ratio for the post hoc test; p.post, Fisher Exact Test post hoc p-value; p.adj.post, false discovery rate (FDR) adjusted Fisher Exact Test post hoc p-value.

Supplementary Table 3. Biomarker Subpopulation Confusion Matrix Metrics and Permutation Test Results for All Metabolites and Ratios of Metabolites.

Direction indicates if the biomarker positive values are greater than or less than the biomarker stratification threshold. The column ‘p.exceedobs’ contains the p-value based on the frequency that the observed performance metrics of sensitivity, specificity, and PMP-ASD were exceeded in 250 iterations of 4-fold cross validation using random permutations of the participants’ diagnoses. The column ‘p.exceedobs_FDR’ is the false discovery rate corrected p-value. The PassMinimum column indicates if the biomarker met minimum diagnostic criteria (TRUE) of sensitivity ≥ 5%, specificity ≥ 95%, and PMP-ASD ≥ 90% or did not (FALSE). Abbreviations: PMP-ASD; percentage biomarker-positive autism spectrum disorder (ASD/(ASD+TYP)).

Supplementary Table 4. Pairwise Biomarker Subpopulations Probabilities of Co-occurrence.

The diagonal contains the number of participants identified in the subpopulation by the column biomarker. The value in the rows is the conditional probability that a participant in the row biomarker is positive given a positive biomaker subpopulation for the column biomarker. Conditional probability = p(Row|Column) = (Column ∩ Row/Column).

Supplementary Table 5. Biomarker Assignment to Biomarker Clusters

Abbreviations: Lac, lactate; Pyr, pyruvate, aKG, alpha-ketoglutarate; Xan, xanthine; Car, carnitine; BCAA, branched chain amino acid; C5DC, glutarylcarnitine; C6, hexanoylcarnitine; C8, octanoylcarnitine; C10, decanoylcarnitine; C16, palmitoylcarnitine. Eta, ethanolamine; Kyn, kynurenine; 4Hyp, 4-hydroxyproline.

Supplementary Table 6. Pairwise Biomarker Cluster Probabilities of Co-occurrence.

The diagonal contains the number of biomarker cluster positive participants identified by the cluster in the column. The value in the rows of the columns containing biomarker clusters is the conditional probability that the cluster in the row is positive given a positive result in the column. Conditional probability = p(Row|Column) = (Column ∩ Row/Column).

Supplementary Table 7. Change in Metabolite Values Between Biomarker Cluster Positive and Cluster Negative Populations.

The cluster biomarker column indicates if the metabolite is present as a ‘numerator’ or ‘denominator’ in one or more of the biomarkers associated with cluster. Metabolites that are not present in a biomarker associated with the cluster are indicated with ‘No’. The column ‘Fold (Pos/Neg)’ contains the fold change values between biomarker cluster positive (Pos) and biomarker cluster negative populations (Neg). The ‘Effect Size’ column contains the Wilcox rank sum test effect size for the comparison between cluster positive and cluster negative. The ‘p’ column contains the Wilcox rank sum p-value between cluster positive (Pos) and cluster negative (Neg) populations. The ‘FDR’ column contains the false discovery rate adjusted p-values. The ‘Pattern Group’ column indicates the biomarker cluster grouping in figure 3.

Supplementary Table 8. Biomarkers Included in the Optimized Test Battery.

The biomarkers and their cluster and pattern group associations of the 42 biomarkers optimized into a test battery. Abbreviations: Lac, lactate; Pyr, pyruvate, aKG, alpha-ketoglutarate; Xan, xanthine; Car, carnitine; BCAA, branched chain amino acid; C5DC, glutarylcarnitine; C6, hexanoylcarnitine; C8, octanoylcarnitine; C10, decanoylcarnitine; C16, palmitoylcarnitine. Eta, ethanolamine; Kyn, kynurenine; 4Hyp, 4-hydroxyproline.

Supplementary Table 9: Partial Spearman Correlation Analysis of Biomarkers Values and Numerical CAMP Behavioral and Phenotypic Data

The column pcor is the partial Spearman correlation coefficient, n is the number of samples without missing data, p.value p-value of the partitial correlation coefficient, p.adj is the false discovery rate corrected p-value.

Supplementary Table 10. Kruskal-Wallis and Wilcox Rank Sum Post Hoc Tests Between Categorical CAMP Behavioral and Phenotypic Data and Biomarker Values

Biomarker, biomarker metabolite or ratio metabolites; Variable, CAMP phenotypic and behavioral data; Description, description of the CAMP phenotypic and behavioral data; n, number of samples in the analysis; df, degrees of freedom; p, Kruskal-Wallis p-value; p.adj, false discovery rate (FDR) adjusted Kruskal-Wallis p-value; effsize, Kurskal-Wallis effect size; response1, dichotomized response 1 used for Wilcoxon rank sum post hoc test, response1.Median, median biomarker value of response 1; response2, dichotomized response 2 used for Wilcoxon rank sum post hoc test; response2.Median, median biomarker value of response 2; response1.n, number of samples with response 1; response2.n, number of samples with response 2; wx.p, Wilcoxson rank sum p-value; wx.padj, false discovery rate (FDR) adjusted Wilcoxson rank sum p-value; Fold, mean response 1 / mean response 2.

Supplementary Table 11. Welch T-Tests Between Numerical CAMP Behavioral and Phenotypic Data and Biomarker Positive and Negative Populations

Biomarker, biomarker metabolite or ratio metabolites; Variable, CAMP phenotypic and behavioral data; Description, description of the CAMP phenotypic and behavioral data; Pos.mean, mean CAMP phenotypic or behavioral data value of the biomarker positive population; Neg.mean, mean CAMP phenotypic or behavioral data value of the biomarker negative population; Fold, fold change Pos.mean/Neg.mean; effsize, Welch T-test effect size; p, Welch T-test p-value; p.adj, false discovery rate (FDR) adjusted Welch T-test p-value.

Supplementary Table 12. Fisher Exact and Post Hoc Test Between Categorical CAMP Behavioral and Phenotypic Data and Biomarker Positive and Negative Populations

Biomarker, biomarker metabolite or ratio metabolites; Variable, CAMP phenotypic and behavioral data; Description, description of the CAMP phenotypic and behavioral data; OddsRatio, conditional Maximum Likelihood Estimate (MLE) of the odds ratio for 2×2 cases; CramerV, Cramer’s V included as a measure of effect size of the association for k x 2 cases; p, Fisher Exact Test p-value; p.adj, false discovery rate (FDR) adjusted Fisher Exact Test p-value; Response, dichotomized response used for Fisher Exact Test post hoc, analysis; OddsRatio.post, conditional Maximum Likelihood Estimate (MLE) of the odds ratio for the post hoc test; p.post, Fisher Exact Test post hoc p-value; N.BiomarkerPositive, number of biomarker positive participants with response; TotalPosI, Total number of biomarker positive participants.

Supplementary Table 13: Partial Spearman Correlation Analysis of Number of Biomarker Clusters and Numerical CAMP Behavioral and Phenotypic Data

Variable, CAMP phenotypic and behavioral data; Description, description of the CAMP phenotypic and behavioral data; n is the number of samples without missing data, p.value p-value of the partial correlation coefficient, p.adj is the false discovery rate corrected p-value.

Supplementary Table 14. Kruskal-Wallis Between Categorical CAMP Behavioral and Phenotypic Data and the number of biomarker clusters

Variable, CAMP phenotypic and behavioral data; Description, description of the CAMP phenotypic and behavioral data; n, number of samples in the analysis; df, degrees of freedom; p, Kruskal-Wallis p-value; p.adj, false discovery rate (FDR) adjusted Kruskal-Wallis p-value; effsize, Kurskal-Wallis effect size.

Supplementary Table 15. Elution Gradient for the Amine Liquid Chromatography Method

A 0.1% formic acid in water; B 0.1% formic acid in acetonitrile

Supplementary Table 16. Elution Gradient for the Plasma Organic Acid Liquid Chromatography Method

A 0.1% acetic acid in 5mM ammonium acetate and B 0.1% acetic acid in acetonitrile

Supplementary Table 17. Elution Gradient for the Unpolar and Microbiome Liquid Chromatography Method

Mobile phase A was 0.2% formic acid in water and mobile phase B was 0.2% formic acid in acetonitrile

**Supplementary References**

1. A. M. Smith, *et al.*, Amino Acid Dysregulation Metabotypes: Potential Biomarkers for Diagnosis and Individualized Treatment for Subtypes of Autism Spectrum Disorder. *Biol. Psychiatry* **85**, 345–354 (2019).
