# Supplementary figures and images for: Metabolomic biomarkers in autism: identification of complex dysregulations of cellular bioenergetics
Source: Front Psychiatry. 2023 Oct 2;14:1249578. doi: 10.3389/fpsyt.2023.1249578 (PMC10622772; doi:10.3389/fpsyt.2023.1249578)

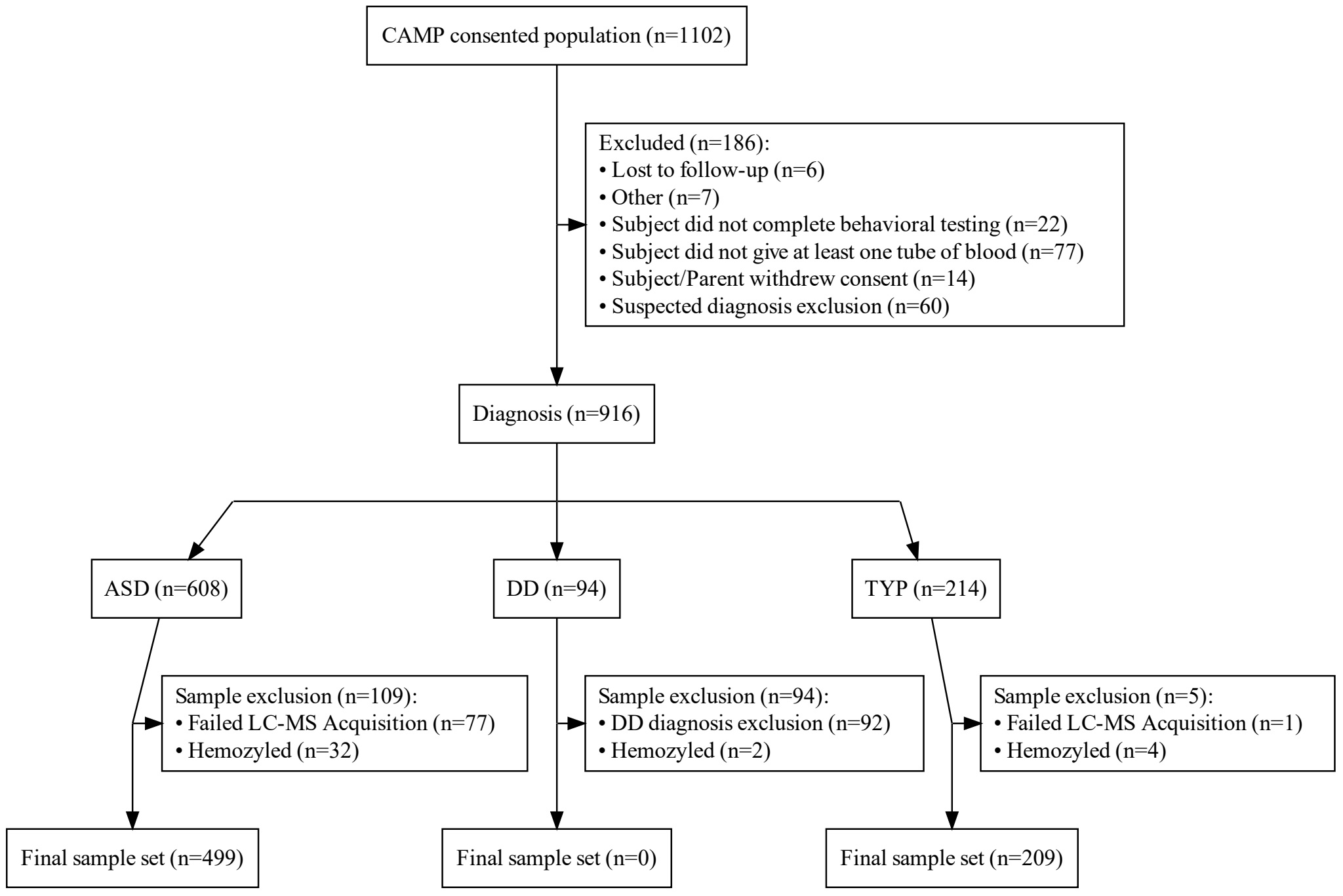

Supplement: Supplementary file 17 [file Image_1.JPEG]

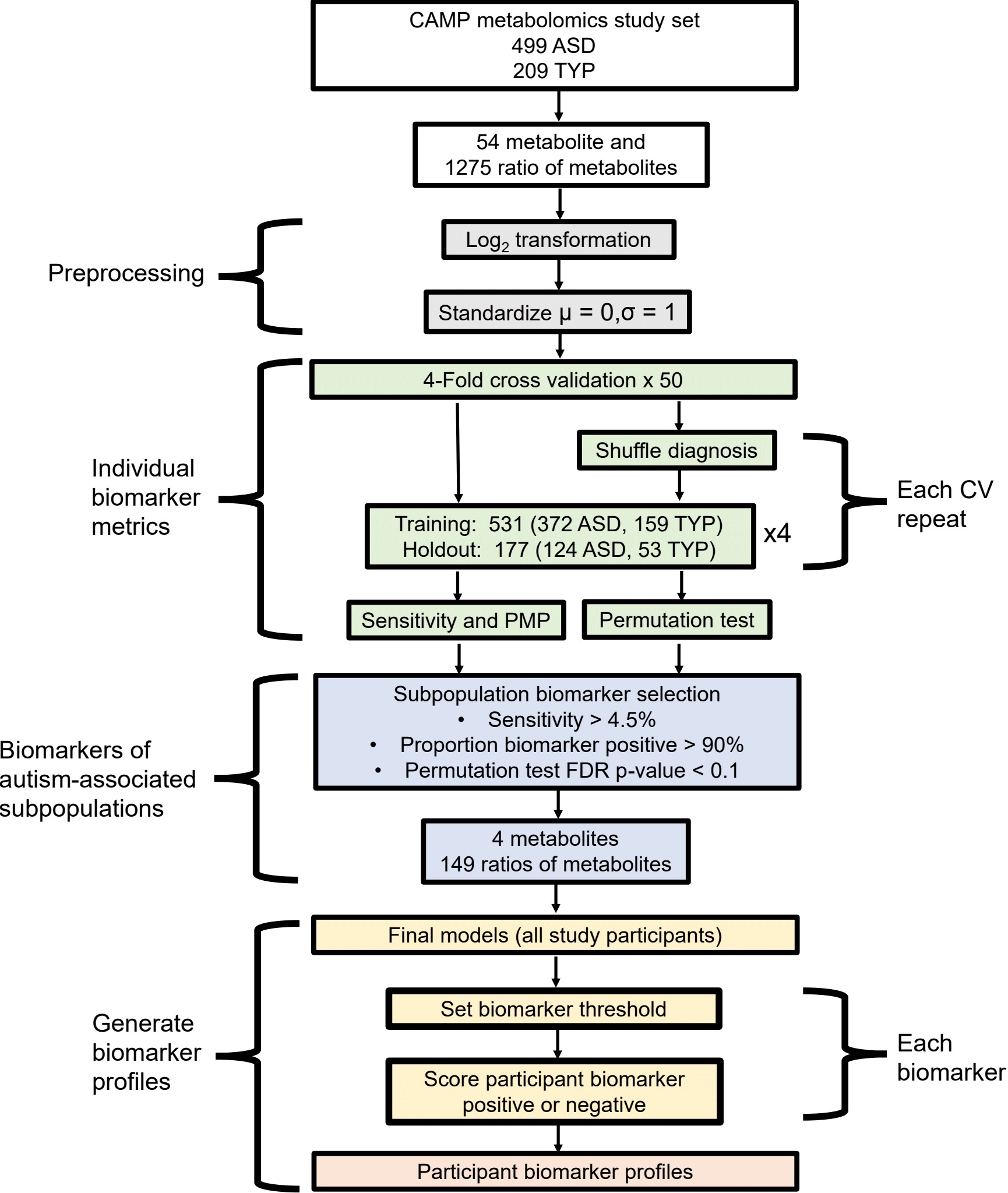

Supplement: Supplementary file 18 [file Image_2.JPEG]

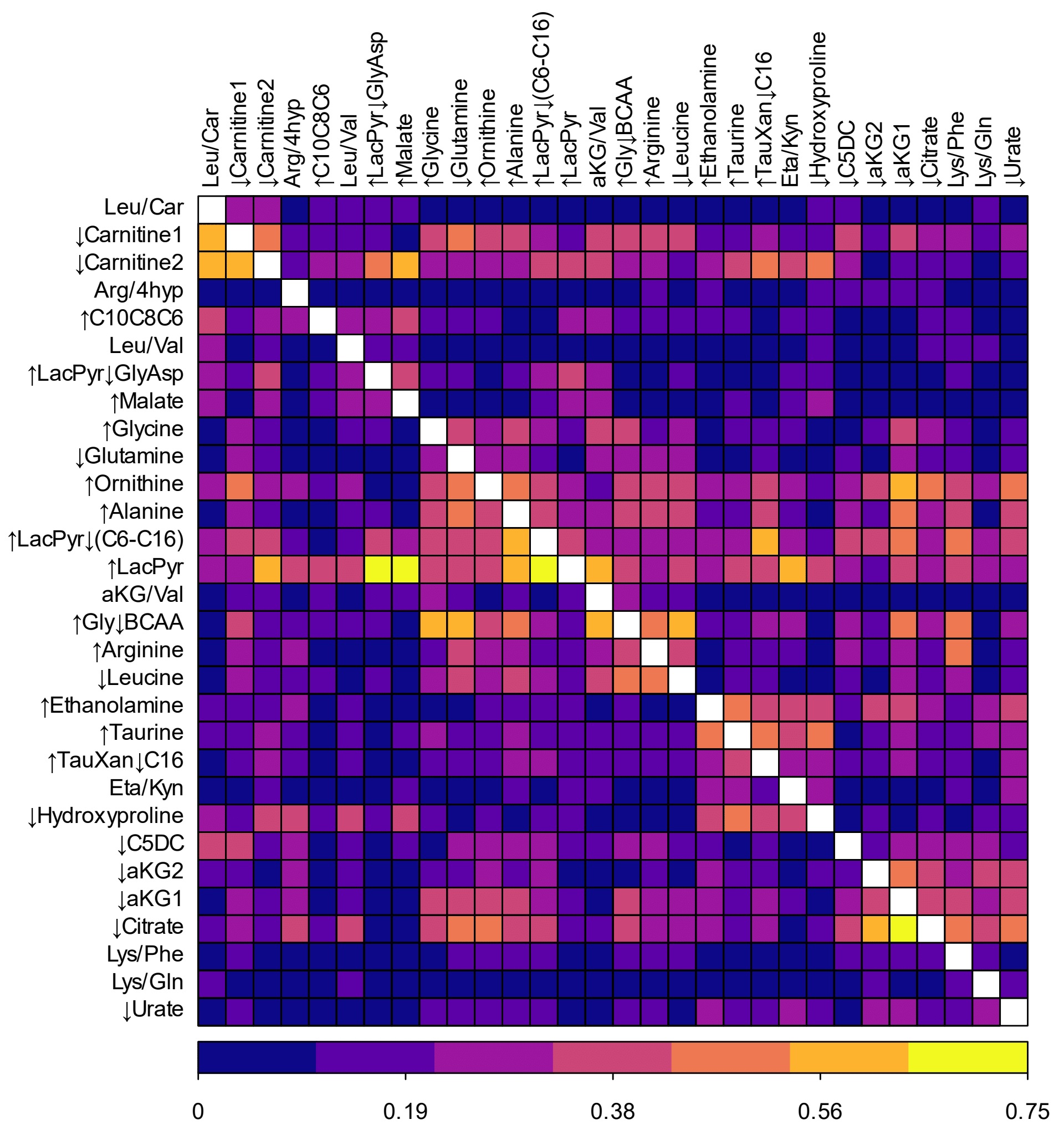

Supplement: Supplementary file 19 [file Image_3.JPEG]
